# Measuring Corneal Thickness with SOCT, the Scheimpflug System, and Ultrasound Pachymetry

**DOI:** 10.5402/2012/869319

**Published:** 2012-09-02

**Authors:** Ilona Piotrowiak, Beata Soldanska, Mateusz Burduk, Bartlomiej J. Kaluzny, Jozef Kaluzny

**Affiliations:** Department of Ophthalmology, Collegium Medicum, Nicolaus Copernicus University, 85-650 Bydgoszcz, Poland

## Abstract

*Background and Objective*. Evaluation of agreement, repeatability, and reproducibility of central and minimal corneal thickness (CCT and MCT) measurements obtained by SOCT, the Scheimpflug system, and ultrasound pachymetry. *Materials and Methods*. 28 eyes of healthy patients were enrolled. Pachymetry measurements were performed with SOCT, the Scheimpflug system, and ultrasound instrument. Each measurement was taken by 3 operators on 3 devices providing a total of 2100 measurements. *Results*. The mean CCT for SOCT, Scheimpflug system, and ultrasound instrument was 537.92, 545.94, and 555.74 *μ*m, respectively, (*P* < .001). The respective mean coefficients of repeatability for CCT were 0.61, 0.82 and 0.80, whereas mean coefficients of interoperator reproducibility for CCT were 0.91, 1.11, and 1.25. *Conclusions*. CCT and MCT measurements show moderate agreement between instruments. The repeatability and interoperator reproducibility of the results obtained by SOCT are somewhat higher. The operator's impact on CCT and MCT measurements is insignificant in all devices.

## 1. Introduction

Central corneal thickness (CCT) measurement plays a major role in diagnostic and therapeutic approaches to corneal pathology and has an important impact on intraocular pressure readings. An ideal method of corneal thickness measurement should be accurate, repeatable, reproducible, and safe, as well as easy and quick to perform. CCT can be assessed by means of many instruments, including specular microscopy, confocal microscopy, ultrasound pachymetry, ultrasound biomicroscopy (UBM), slit-scanning corneal topography, the Scheimpflug system, optical biometry, and spectral optical coherence tomography (SOCT). Ultrasound pachymetry, which used to be a gold standard for measuring corneal thickness, is a contact method where the place of measurement is strongly dependent on the operator [1]. Therefore, other noninvasive methods have gained popularity; nevertheless, the accuracy of some of these methods has not been thoroughly examined. The most important features of a measuring technique are accuracy and precision. Because the true value of the corneal thickness of a particular eye is unknown, it is impossible to calculate the accuracy of measurements. Therefore, the agreement between new devices and an instrument considered to be a gold standard should be used. The precision of measurements may be stratified into repeatability and reproducibility. Repeatability is defined as the variability of results obtained from one object in the same measurement conditions (time, instrument, technique, place, and operator), while reproducibility is the variability of results obtained from one object using the same device in different measurement conditions [2]. In this study, we assessed the agreement, repeatability, and interoperator reproducibility of CCT and minimal corneal thickness (MCT) measurements obtained with two modern noncontact devices (SOCT and the Scheimpflug system), as well as conventional ultrasound instrument.

## 2. Materials and Methods

The study was conducted in the Department of Ophthalmology, Collegium Medicum, Nicolaus Copernicus University in Bydgoszcz, Poland, after written informed consent was provided by the participants following the Bioethics Committee guidelines. Eyes with apparent corneal pathologies that might influence corneal thickness or structure were excluded from the study. The corneal thickness was evaluated in 28 eyes (14 patients). The mean age of the study group participants was 36.86 ± 7.95 years (range, 24–50 years). The group included 11 female (78.6%) and 3 male (21.4%) participants.

CCT of every eye was measured by three operators, consecutively. Each operator took five measurements with each instrument (RTVue 100, Optovue; Pentacam HR, Oculus; Pachette 3, DGH). The measurements were taken the same day with ten-minute intervals between operators, giving a total of 2100 measurement.

The way corneal thickness is evaluated differs among the three instruments. The RTVue-100 FD is based on SOCT; it is a frequency-domain OCT utilizing an 840 nm wavelength. A cornea adapter module (CAM) was used to visualize and measure anterior chamber parameters. The duration of a single examination consisting of 8 tomograms is 0.3 sec. The Pentacam HR is a rotating Scheimpflug camera that provides 25 tomograms of the anterior segment of the eye in three dimensions in less than 2 sec. It utilizes monochromatic slit light of 475 nm wavelength. The Pachette 3 is an ultrasound pachymeter that provides A scans. It is equipped with a 20 MHz probe. The time of an examination consisting of 10 single measurements usually does not exceed 3 sec. Because ultrasound pachymetry is the only contact method used in this study, ultrasound measurements were taken last to avoid cornea changes caused by probe application that might influence the two other measurements.

Agreement was assessed on the basis of discrepancy between measurements taken with different instruments. The variability was described by repeatability and reproducibility of the results. Repeatability of CCT and MCT measurements was estimated by the coefficient of repeatability which is defined as the standard deviation divided by the mean result. The lower the coefficient of repeatability, the more repeatable the measurements were. Interoperator reproducibility was estimated by comparison of the operators' results in pairs (operator 1 with operator 2, operator 2 with operator 3, and operator 1 with operator 3) for all the devices consecutively. The coefficient of reproducibility was then calculated as the SD of the differences between the pairs of measurements obtained by each pair of operators, divided by the average of the means of each pair of measurements. As with the coefficient of repeatability, the lower the coefficient of reproducibility, the more reproducible the measurements were. Conditions were standardized by using the same method, the same patient, the same device, and the same operator (pairs of operators) in short-time intervals. Subsequent multifactorial analysis of variance was conducted using Statistica 6.0 PL software.

### 2.1. Limitations of the Study

The study revealed some instrument-dependent limitations. The RTVue 100 FD resulted in some MCT readings that were higher than the CCT readings. Additionally, an archiving error occasionally occurred in a single measurement. In both cases, the data was ignored and the measurement was retaken. Pentacam HR measurements often had to be repeated because of an incorrect result caused by fixation loss, blinking, or incorrect head position. Pachette 3 was the only contact method used in our study and it required topical anesthesia. The subjective choice of measurement place is an inherent disadvantage of this technology.

## 3. Results

### 3.1. Agreement

**Figure 1:**
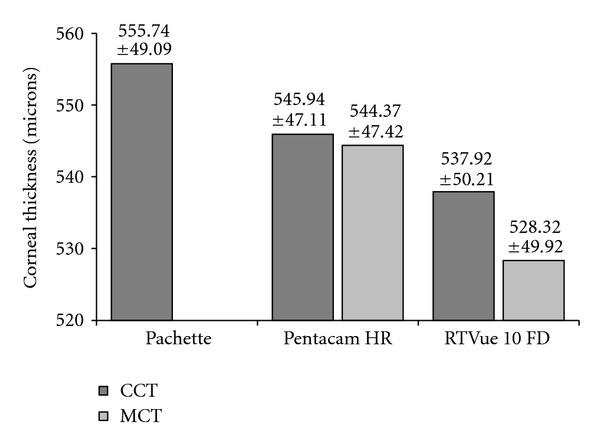
Mean CCT and MCT values obtained with 3 different devices.

The mean values of CCT and MCT obtained by the RTVue 100 FD, Pentacam HR, and Pachette 3 are shown in Figure 1.

**Figure 2:**
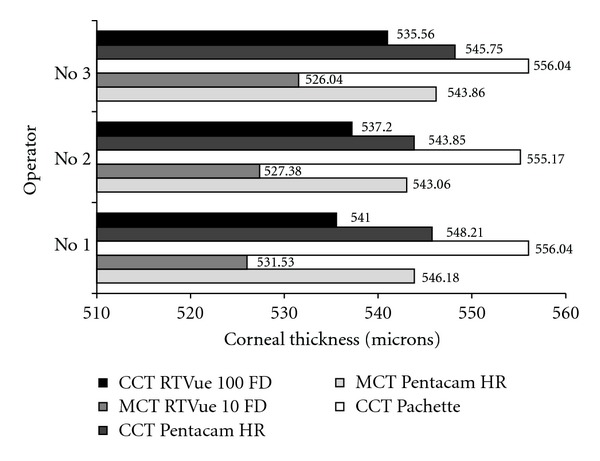
Mean CCT and MCT obtained with different instruments and operators.

The differences between the devices are statistically significant (*P* < .001 for both CCT and MCT). The CCT results obtained with the RTVue 100 FD and Pentacam HR are 3.21% and 1.76%, respectively, lower than with the Pachette. The MCT results obtained with the RTVue 100 FD are 2.95% lower than with the Pentacam HR.  Figure 2 depicts the mean CCT and MCT with respect to each instrument and operator.

### 3.2. Repeatability

The mean standard deviations for a series of 5 CCT and MCT measurements of one eye taken by one operator are summarized in Table 1.

The coefficients of repeatability for CCT and MCT measurements for different instruments and operators are summarized in Table 2.

The mean coefficient of CCT repeatability was 0.61 for the RTVue 100 FD, 0.82 for the Pentacam HR, and 0.80 for the Pachette 3. The mean coefficient of MCT repeatability was 0.41 for the RTVue 100 FD and 0.81 for the Pentacam HR. The RTVue 100 FD demonstrated significantly higher repeatability of CCT and MCT measurements when compared with the Pentacam HR and Pachette 3 (*P* < .001).

### 3.3. Reproducibility

Analysis of the mean CCT and MCT, taking into consideration both the instrument and the operator, proved that the differences between mean results are significant (*P* < .001; Figure 2). Statistical analysis revealed that the bifactorial differences are due to the instrument and not the operator. All operators obtained the same tendency of results (i.e., the highest from the Pachette 3, followed by the Pentacam HR and RTVue 100 FD). The coefficients of interoperator reproducibility are shown in Table 3.

The mean coefficient of CCT reproducibility was 0.91 for the RTVue 100 FD, 1.11 for the Pentacam HR, and 1.25 for the Pachette 3 (*P* < .003). The mean coefficient of MCT reproducibility was 0.91 for the RTVue 100 FD and 1.27 for the Pentacam HR (*P* < .003).

## 4. Discussion

Corneal thickness measurement is an examination of increasing clinical importance. It is useful for diagnosing and monitoring conditions such as corneal edema or ectasia [3]. Corneal thickness plays an important role in evaluation of intraocular pressure and glaucoma progression risk in patients with ocular hypertension as thinner corneas may lead to the underestimation of intraocular pressure [3–7]. Additionally, corneal thickness measurement is necessary to determine and plan the type and extent of corneal refractive surgery [3, 6].

Ultrasound pachymetry has been considered the gold standard for corneal thickness measurement for many years [8–10]. However, many other methods can be used, including Scheimpflug systems and SOCT, which have the great advantage of noncontact. These methods use different measurement technologies and give different results. According to different researchers, CCT measurement obtained with standard ultrasound pachymetry varies from 542 to 550 *μ*m [1, 10–12]. In our hands, the mean CCT measured by the Pachette 3 was somewhat higher: 555.74 ± 49.09 *μ*m. With the use of the Pentacam HR, the results of CCT by different researchers were between 544 and 552 *μ*m [1, 10]; our result of 545.94 ± 47.11 *μ*m is within the previously reported range. The mean CCT measured by different types of OCT instruments was previously reported within the range of 523–527 *μ*m [13, 14]. In our hands, the mean CCT measured with the RTVue 100 FD was 537.92 ± 50.21 *μ*m. The mean CCT measurements from the Pachette 3 are 9.8 *μ*m higher than those from the Pentacam HR, and 17.82 *μ*m higher than those from the RTVue 100 FD; the differences are statistically significant. These results remain in accordance with other studies in which the Pentacam HR and SOCT measurements were lower than ultrasound pachymetry measurements, on average, by 6.0–9.8 *μ*m and 11.64–49.4 *μ*m, respectively [1, 9, 13–19]. One possible reason for the difference may be the use of topical anesthetics to take a contact measurement with the ultrasound pachymeter which may cause corneal epithelial edema resulting in overestimation of the results [15]. Additionally, the subjective choice of the place of measurement can lead clinicians to obtain results from paracentral regions of the cornea. However, there are also studies [1, 20] showing an opposite relation, in which the results from ultrasound pachymetry are lower in comparison to other methods. According to the authors, ultrasound pachymetry causes tear film dislocation and epithelium compression, resulting in CCT measurements that are lower by 7–30 *μ*m. We hypothesize that CCT readings with ultrasound pachymeters may depend on which model of the instrument is used.

A pivotal feature of the modern pachymeter is high repeatability of results. When comparing mean standard deviations of CCT and MCT measurements in a series of 5 measurements from the same patient by different devices regardless of operator, the RTVue 100 FD shows the lowest values (Table 1). Repeatability may also be expressed as the coefficient of repeatability, defined as the standard deviation divided by the mean result [21]. The mean respective coefficients of CCT repeatability were 0.61%, 0.82%, and 0.80% for the RTvue 100 FD, Pentacam HR, and Pachette 3. The mean respective coefficients of MCT repeatability were 0.41% and 0.81% for the RTVue 100 FD and Pentacam HR. In a study by Barkana et al. [9], the coefficient of repeatability was 0.74% for Pentacam HR and 0.71% for ultrasound pachymetry. According to the authors, such repeatability makes this examination practically independent of the operator and enables reliable measurement in just one reading. Muscat et al. [22] assessed the repeatability of OCT (Humphrey-Zeiss Medical Systems) and the average coefficient of repeatability was 2% (1.76% for horizontal scans and 2.32% for vertical scans) which is, according to the authors, sufficient to obtain and monitor corneal thickness in a reliable and useful way. Similar results were shown by Shaheeda et al. [3] for Visante OCT with a coefficient of repeatability of 2% in healthy eyes and 3% in keratoconic eyes. Its higher value compared with our results may be due to using a double-standard deviation to calculate the coefficient. In another study by de Sanctis et al. [23] conducted on keratoconic eyes using ultrasound pachymetry and the Scheimpflug system, the coefficient of repeatability for the Allergan-Humphrey 850 was twice of that for the Pentacam HR. Our results confirm that all of the instruments evaluated in this study provide measurements with clinically sufficient repeatability and the RTVue 100 FD performs slightly better in this area.

We used reproducibility to assess the operator's impact on the results. In the present study, we compared the results of pairs of operators taking measurements with every device, consecutively. The coefficient of reproducibility was then calculated as the standard deviation of the difference of the two measurements divided by the average of each pair of results. The average coefficients of reproducibility for CCT measurements taken by three operators using the RTvue 100 FD, Pentacam HR, and Pachette 3 were 0.91%, 1.11%, and 1.25%, respectively. For MCT measurements obtained using the RTVue 100 FD and Pentacam HR, the coefficients of reproducibility were 0.91% and 1.27%, respectively. In the study by Muscat et al. [22], OCT demonstrated a much lower coefficient of reproducibility of 0.18%, which, at least in part, may be due to the comparison of only one pair of operators. The coefficient of reproducibility between a pair of operators estimated by Shaheeda et al. [3] for Visante OCT measurements was 2% in healthy eyes and 4% in keratoconic eyes; however, similarly to the coefficient of repeatability calculation, the double-standard deviation was used. Barkana et al. [9] assessed reproducibility between two operators for the Pentacam HR and their published coefficient of reproducibility (1.10%) was similar to ours. A study by De Sanctis et al. [23] on ultrasound pachymetry showed that differences between measurements taken by three operators were higher than those between measurements of one operator which, according to the authors, proves the great impact of an operator to the results. Subjective estimation of the center of the cornea, the technique of placing a probe, and its subsequent perpendicular alignment may all contribute to the discrepancy of measurements. In the case of the Scheimpflug system, the reproducibility and repeatability of results are similar; according to the authors, this similarity is due to higher automatization of the examination which depends primarily on correct patient position and gaze, and only to a lesser extent on the operator. In the present study, none of the results obtained from different instruments depended significantly on the operator.

## 5. Conclusions

CCT and MCT measurements demonstrate moderate agreement between instruments. Therefore, different technologies cannot be used interchangeably without using correcting coefficients. The repeatability and interoperator reproducibility of the measurements obtained by the RTVue 100 FD are somewhat higher than those of other systems. The operator's impact on CCT and MCT measurements is insignificant in all devices.

## Figures and Tables

**Table 1:** Mean standard deviation for CCT and MCT measurements obtained with different instruments.

Instrument	Mean standard deviation	
	CCT	MCT
RTVue 100 FD	±3.29	±2.17
Pentacam HR	±4.47	±4.41
Pachette 3	±4.44	—

**Table 2:** Coefficient of repeatability of CCT and MCT measurements for different instruments and operators.

Coefficient of repeatability	Operator	System		
		RTVue 100 FD	Pentacam HR	Pachette 3
CCT	1	0.63%	0.81%	0.66%
	2	0.60%	0.85%	0.88%
	3	0.61%	0.80%	0.85%

MCT	1	0.42%	0.81%	—
	2	0.41%	0.89%	—
	3	0.40%	0.73%	—

**Table 3:** Coefficient of interoperator reproducibility of CCT and MCT measurements for different instruments and operators.

Coefficient of reproducibility	Operator	System		
		RTVue 100 FD	Pentacam HR	Pachette 3
CCT	1 and 2	0.92%	1.00%	1.25%
	1 and 3	1.08%	1.27%	1.05%
	2 and 3	0.72%	1.06%	1.44%

MCT	1 and 2	1.00%	0.95%	—
	1 and 3	1.16%	1.55%	—
	2 and 3	0.58%	1.31%	—
